# The Origin of Syphilis

**Published:** 1905-07

**Authors:** 


### *EDITORIAL NOTES AND COMMENTS.*

The Business office of THE JOURNAL-RECORD is No. 1007-1008 Century Building.

The Editorial office is 318-319-320 The Prudential.

Address all Business communications to Dr. M. B. Hutchins, Mgr.

Make remittances payable to THE ATLANTA JOURNAL-RECORD OF MEDICINE.

On matters pertaining to the Editorial and Original Communications address Dr Bernard Wolff, Atlanta.

Reprints of original articles will be furnished at cost price. Requests for the same should always be made *on the manuscript.*

We will present, post-paid, on request, to each contributor of an original article, twenty (20) marked copies of THE JOURNAL-RECORD containing such article.

### THE ORIGIN OF SYPHILIS.

To consider the origin of disease is to consider the origin of man, for the two have waged the unequal contest since heaven and earth sprang out of chaos. When man first made his advent into this vale of tears he encountered hostile influences in manifold guises divinely adjusted to the need of peopling the world with those fittest to survive. The weakest succumbed first, the stronger lingered long enough to beget those who by a heritage of strength were destined to add a link to the racial chain—then they, too, yielded to the inevitable. These things were achieved by disease, not disease in the abstract but such concrete actualities as pneumonia, tuberculosis, syphilis and so on throughout the whole unhappy list. There is no reason to presume for an instant that the same busy enemies did not exist to destroy the skin-clad brother to the wolf of whom we catch half imagined glimpses in the rolling darkness that covered the earth's youth as in these latter days when a little knowledge has made us so wise in our own conceit. All that we know has been dug out of the store-house of the ages or rescued from the rubbish-heap.

The revelations of science, the illumination of discovery are not creative terms. They merely mean that now we may see what was before invisible.

With this view—and who can gainsay it—it is absurd to search for the origin of disease in its liberal historical sense—and so with syphilis. The canard that it is of American origin is as ridiculous as some of the qualifying geographical adjectives which it has gathered unto itself during its triumphant march through the ages. Beyond peradventure primitive man sought seclusion to scrutinize his chancre much in the same way, but with different emotions, as does the conscience-stricken wretch of to-day examine his genital lesion with tremulous apprehension. The following is, however, the accepted view of the origin of syphilis and is given for what it may be worth:

ORIGIN OF SYPHILIS.

Although the introduction of syphilis among Europeans is of comparatively recent date, its history is shrouded so mysteriously that we can only guess at its origin. Even the etymology of the word is obscure. Some derive it from the Greek "syn" with, and "philos" love, but there is much doubt about this. The word, as far as can now be learned, was first used in 1530, as the title of a poem by Hieronymis Fracastorius, an Italian physician, and a man of such great culture and elegance of Latin style that he almost merited his epithet of "The Divine."

There is a fable that the disease dated from the siege of Naples (1494), when the besieged Spanish garrison, comprising some of the crew of Columbus, infected the French army, who in turn rapidly scattered it throughout Europe; hence the common name, morbus neapolitanus, after the place of its birth. Probably both names show only the racial antipathies of mud-throwing patriots.

It is interesting to note, in view of some more modern ideas of syphilis, that Paracelsus attributed the origin to the intercourse of a leper with a prostitute, and that Fallopius informed the professional world that the original epidemic of the "French disease" came from wine, which the Spaniards, in the siege of Naples, had poisoned with the blood of a leprous soldier. When the besieging and victorious French took possession of the city, they drank freely of the luscious wine, and soon after developed typical symptoms of venereal disease. Even our own chancellor, Lord Bacon, averred that in this famous siege the French ate human flesh which had been prepared from men killed in Barbary, where the people consume large quantities of fish. And so the theories of the close relationship between syphilis, leprosy and fish are not at all modern.

There is no doubt that venereal diseases contracted from individuals of a remoter race, are some markedly severe and much more destructive in their type than those contracted from persons of the same nationality. The chancres of the wharf districts of large cities are always bad because the females of those localities enjoy continuous patronage of the foreign sailors and refugees—the dregs of other countries. And so there may be a grain of truth in the fable to which we referred. It may be that syphillis, in a very mild form, was comparatively common all over Europe, but that the fresh inoculation, brought back from America, and so freely scattered about during the siege of Naples, gave a new vigor to the disease, which attracted the attention of physicians, and caused them to mistake it for a new infection.—*Canadian Practitioner and Review.*

---

The Secretary of the Georgia State Commission on Tuberculosis, which was created by a joint resolution of the General Assembly at its session of 1904, begs leave to submit this report of the work of the Commission. BERNARD WOLFF, M.D., Sec'y.

